# Supplementary material for: Classification of virulence factors based on dual-channel neural networks with pre-trained language models
Source: PLoS One. 2026 Jan 5;21(1):e0340194. doi: 10.1371/journal.pone.0340194 (PMC12768247; doi:10.1371/journal.pone.0340194)
Supplement: S1 File — (DOCX) [file pone.0340194.s001.docx]

**Supplemental Document 1:**

**Distribution of pLDDT and Correlation with Model Performance**

The Predicted Local Distance Difference Test (pLDDT) [1] is a confidence metric used to evaluate the local accuracy of individual residues in protein structure predictions. It employs a quantitative scoring system ranging from 0 to 100: regions with scores above 90 indicate high-confidence predictions (highly reliable structures), scores between 70 and 90 represent medium confidence (reasonable local topology but potentially deviant side-chain orientations), scores between 50 and 70 suggest low reliability (generally correct backbone folding but requiring cautious interpretation of atomic positions), and scores below 50 indicate potentially unreliable structural domains. Based on this, the mean pLDDT score across all residues was used in this study as the core metric for assessing the overall confidence of protein structure predictions.


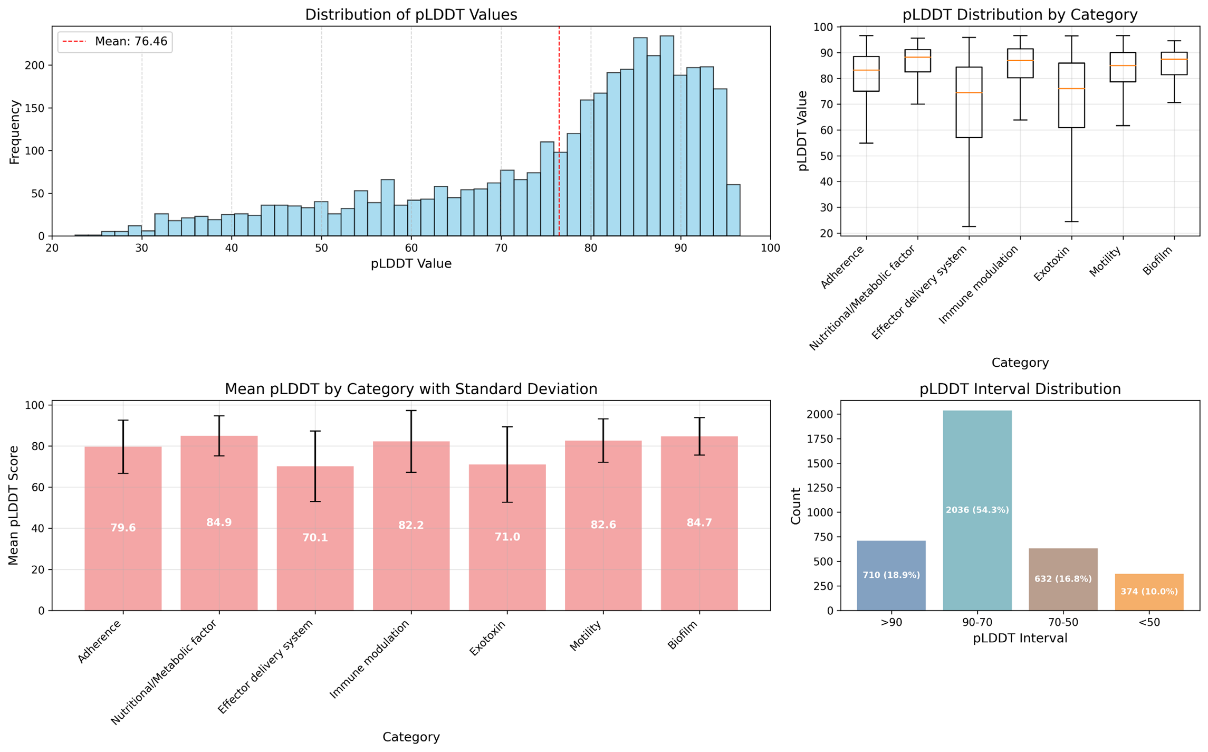


**S1 Fig. Multi-perspective statistical analysis of pLDDT scores.**

Through multidimensional visualization analysis, we revealed the distribution characteristics of pLDDT values in protein structures and their heterogeneity across categories. S1 Fig, comprising a global frequency distribution histogram and a interval distribution proportion bar chart, shows that pLDDT values follow a right-skewed unimodal distribution, with the frequency peak concentrated in the 80–90 interval (mean = 76.46). The data are primarily distributed within the 50–100 range (>90 interval accounting for 18.9%, 70–90 interval accounting for 54.3%), indicating that most prediction results exhibit relatively high reliability. However, low-confidence data (<50, accounting for 10.0%) still require careful validation. Fig 1, which includes inter-category boxplots and bar charts of category means and dispersion, jointly reveals differences in prediction reliability across categories: among these, the *Biofilm* category exhibits the highest median, with the box tightly concentrated in high-value intervals, a mean of 84.7, and the smallest standard deviation, suggesting optimal reliability in its structural predictions. In contrast, the "*Effector delivery system*" category shows the lowest median, the widest box span, a mean of only 70.1, and a higher standard deviation compared to other categories, indicating considerable fluctuation in its pLDDT values. The *Exotoxin* category similarly demonstrates a broad distribution range, while the "*Nutritional/Metabolic factor*" category exhibits a relatively narrow box and a high mean of 84.9, reflecting greater prediction stability. In summary, the reliability of protein structure predictions is generally medium to high, but there are certain differences in pLDDT value distributions across categories.

Given that this study employed the ESMFold model for protein structure prediction, whose output structures may deviate to some extent from the true structures, it is necessary to explore the potential relationship between pLDDT and misclassification by the classification model. To analyze the correlation between pLDDT scores and misclassification in this model, we calculated both Pearson and Spearman correlation coefficients between the mispredicted label vector of the PLM-GNN and the pLDDT score vector. The results show that the p-value for the Pearson correlation coefficient is 0.1519, and the p-value for the Spearman correlation coefficient is 0.0987, both exceeding the significance level of 0.05. This indicates that the lack of a significant correlation between the pLDDT score and classification errors demonstrates the strong robustness of our PLM-GNN model. We hypothesize that this may be attributed to the overall high pLDDT values in the dataset, with a small number of low-pLDDT samples having minimal impact on the overall model performance.

**Reference**

1.Tunyasuvunakool K, Adler J, Wu Z, et al. Highly accurate protein structure prediction for the human proteome[J]. Nature, 2021, 596(7873): 590-596. doi: https://doi.org/10.1038/s41586-021-03828-1
