# Supplementary material for: Classification of virulence factors based on dual-channel neural networks with pre-trained language models
Source: PLoS One. 2026 Jan 5;21(1):e0340194. doi: 10.1371/journal.pone.0340194 (PMC12768247; doi:10.1371/journal.pone.0340194)
Supplement: S2 File — (DOCX) [file pone.0340194.s002.docx]

**Supplemental Document 2:**

**Detailed Derivation of Geometric Graph Features**

We set 15 Å as the default radius to construct the radius graph. The core of all geometric features is a local right-handed coordinate system defined for each residue *i*, based on its backbone atoms (*Cα, C, N*). The vectors for each residue *i* are defined as follows:

$$u_{i}=\text{ }N_{i}-\text{ }C\alpha_{i},v_{i}=\text{ }C_{i}-\text{ }C\alpha_{i}\text{，}$$

Next, the orthonormal basis vectors are computed. The first basis vector *bᵢ* is the normalized vector orthogonal to the plane spanned by *uᵢ* and *vᵢ*; then, *nᵢ* is the normalized projection of *vᵢ* onto the plane perpendicular to *bᵢ*. Finally, *bᵢ*, *nᵢ*, and their cross product together form a right-handed orthonormal coordinate system. The three basis vectors and the corresponding rotation matrix are expressed as follows:

$$b_{i}=\frac{u_{i}\times v_{i}}{\left\|u_{i}\times v_{i}\right\|}\text{，}$$

$$n_{i}=\frac{v_{i}-(v_{i}\cdot b_{i})b_{i}}{\left\|v_{i}-(v_{i}\cdot b_{i})b_{i}\right\|}\text{，}$$

$$Q_{i}=[b_{i},n{}_{i},b_{i}\times n_{i}]\text{，}$$

We then proceed to node feature extraction. First, dihedral and bond angles (*ϕ, ψ, ω, α, β, γ*) are computed using standard biochemical libraries (Biopython) based on the atomic positions of residue i and its adjacent residues. The sine and cosine values of each angle are used to avoid periodicity issues. Next, Radial Basis Function (RBF) embedding is applied to intra-residue distances: for 10 specified atom pairs (e.g., *Cα–N*, *Cα–C, R–O*), the Euclidean distance *‖A − B‖* is calculated. Each distance is encoded into a 16-dimensional vector using a RBF kernel expansion:

$$RBF(d)=[\exp(-\gamma\left\|d-\mu_{1}\right\|^{2}),\exp(-\gamma\left\|d-\mu_{2}\right\|^{2}),...,\exp(-\gamma\left\|d-\mu_{16}\right\|^{2})]\text{，}$$

where *μ_1_​,...,μ_16_*​ are centers equally spaced over the range of expected distances (e.g., 0Å to 15Å).

Next, for node orientation, for each of the four key atoms *V (N, C, O, R)* in residue i, the vector from *Cαᵢ* to *V* is computed and projected into the local frame *Qᵢ*, resulting in a normalized, rotation and translation-invariant direction vector as shown below:

$$direction=Q_{i}^{T}\cdot\frac{(V-C\alpha_{i})}{\left\|V-C\alpha_{i}\right\|}\text{，}$$

Each atom corresponds to a 3D vector, and the four vectors are concatenated to form a 12-dimensional feature. Finally, we extract a pretrained 1024-dimensional feature per node using ProtT5. These features are then concatenated to form the final node representation.

We then proceed to edge feature extraction, which describes the geometric relationship between two residues *i* and *j*. First, for inter-residue distance encoding, 25 inter-residue atom pairs (e.g., *Nᵢ–Nⱼ, Nᵢ–Cαⱼ, Rᵢ–Oⱼ*) are selected, and the Euclidean distance ‖*Aᵢ − Dⱼ*‖ is computed for each pair. Each distance is encoded into a 16-dimensional vector using a RBF kernel expansion (same method as above), resulting in a final 400-dimensional feature vector. Next, for edge orientation, which describes the direction from residue *i*’s *Cα* to an atom in residue *j*, the vector *Dⱼ − Cαᵢ* is calculated for each atom *D* in residue *j* (where *D ∈ {Nⱼ, Cαⱼ, Cⱼ, Oⱼ, Rⱼ}*). This vector is normalized and then projected into the local frame *Qᵢ* of residue *i* to obtain a normalized direction vector. Each atom corresponds to a 3D vector, and the final concatenated feature—with dimensionality corresponding to the number of atom pairs—is formed as follows:

$$direction_{i\to j}=Q_{i}^{T}\cdot\frac{(D_{j}-C\alpha_{i})}{\left\|D_{j}-C\alpha_{i}\right\|}\text{，}$$

We repeat this process from residue *j*'s perspective for symmetry, resulting in a 30-dimensional feature.

Next, for edge orientation, which describes the relative rotational relationship between the local frames of residue *i* and residue *j*, the rotation matrix aligning frame *i* to frame *j* (i.e., *Q_i_^T^Q_j_*) is computed. This rotation matrix is then represented as a 4-dimensional unit quaternion q using a standard matrix-to-quaternion conversion algorithm. Quaternions provide a non-singular representation of rotation. Finally, for edge positional encoding, we extend the Transformer-style positional encoding method to geometric graph structures. Based on the vector between *Cα_i_* and *Cα_j_*, sine and cosine functions of different frequencies are applied to generate a 16-dimensional feature vector that uniquely encodes the spatial geometric relationship between residues. The above features are concatenated to form the final edge feature representation used in our model.
