# Supplementary material for: Classification of virulence factors based on dual-channel neural networks with pre-trained language models
Source: PLoS One. 2026 Jan 5;21(1):e0340194. doi: 10.1371/journal.pone.0340194 (PMC12768247; doi:10.1371/journal.pone.0340194)
Supplement: S3 File — (DOCX) [file pone.0340194.s003.docx]

**Supplemental Document 3:**

**Supplemental Figures and Tables**

**Supplemental Figures**


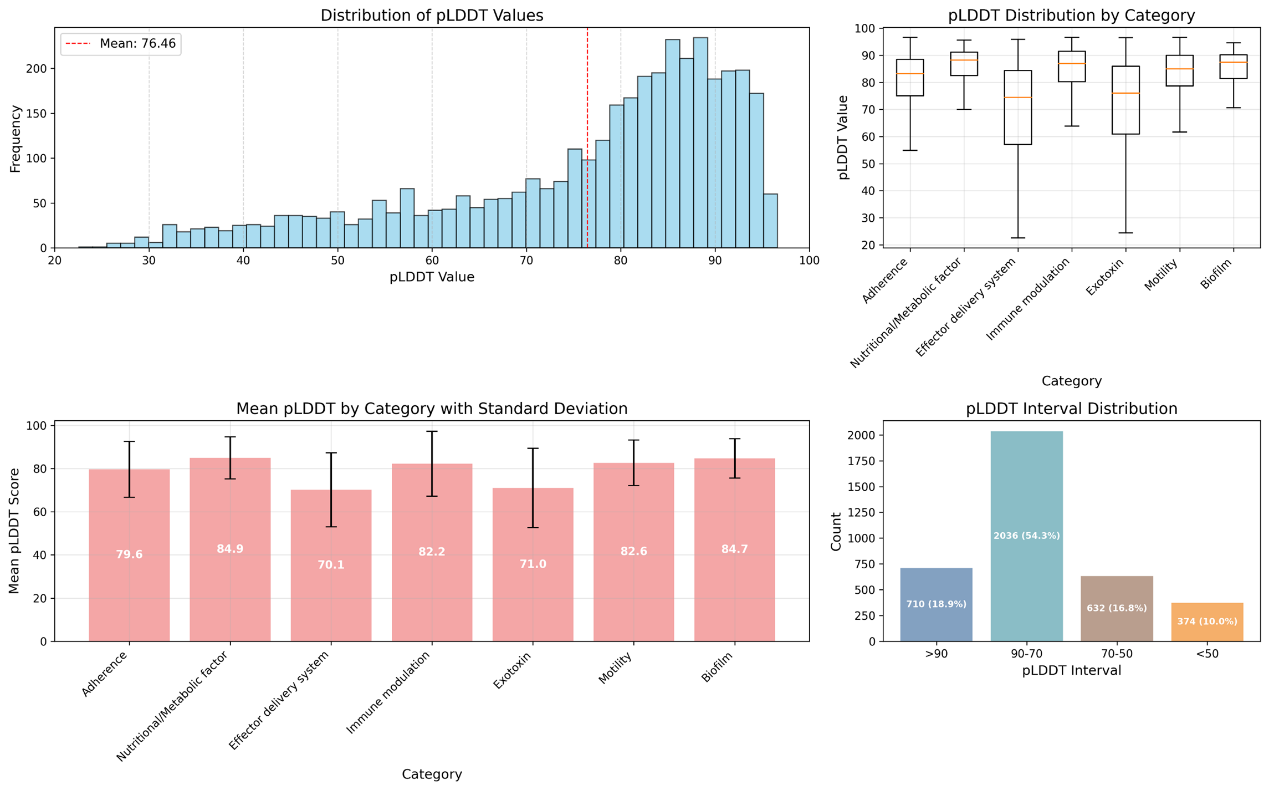


**S1 Fig. Multi-perspective statistical analysis of pLDDT scores.**


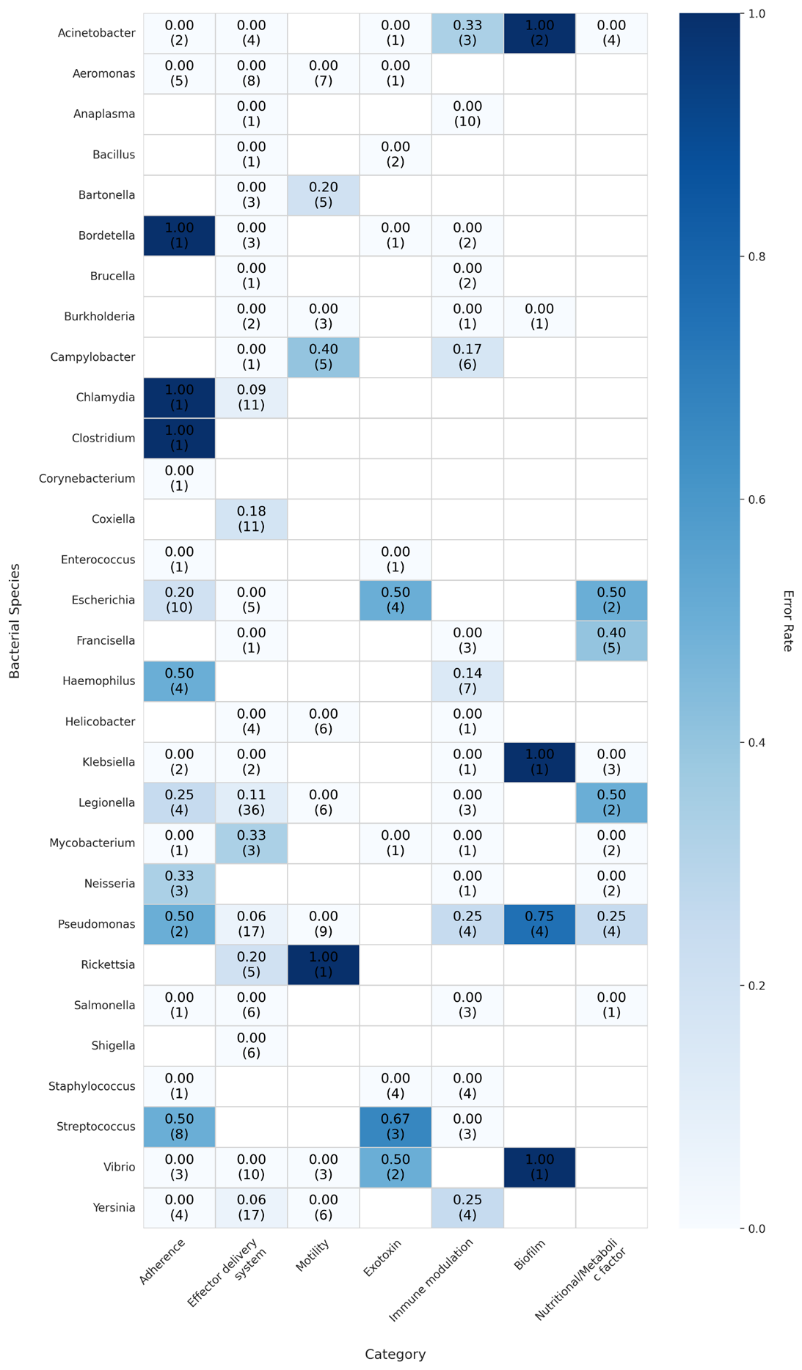


**S2 Fig. Error rate heatmap of bacterial genera by VF category.**


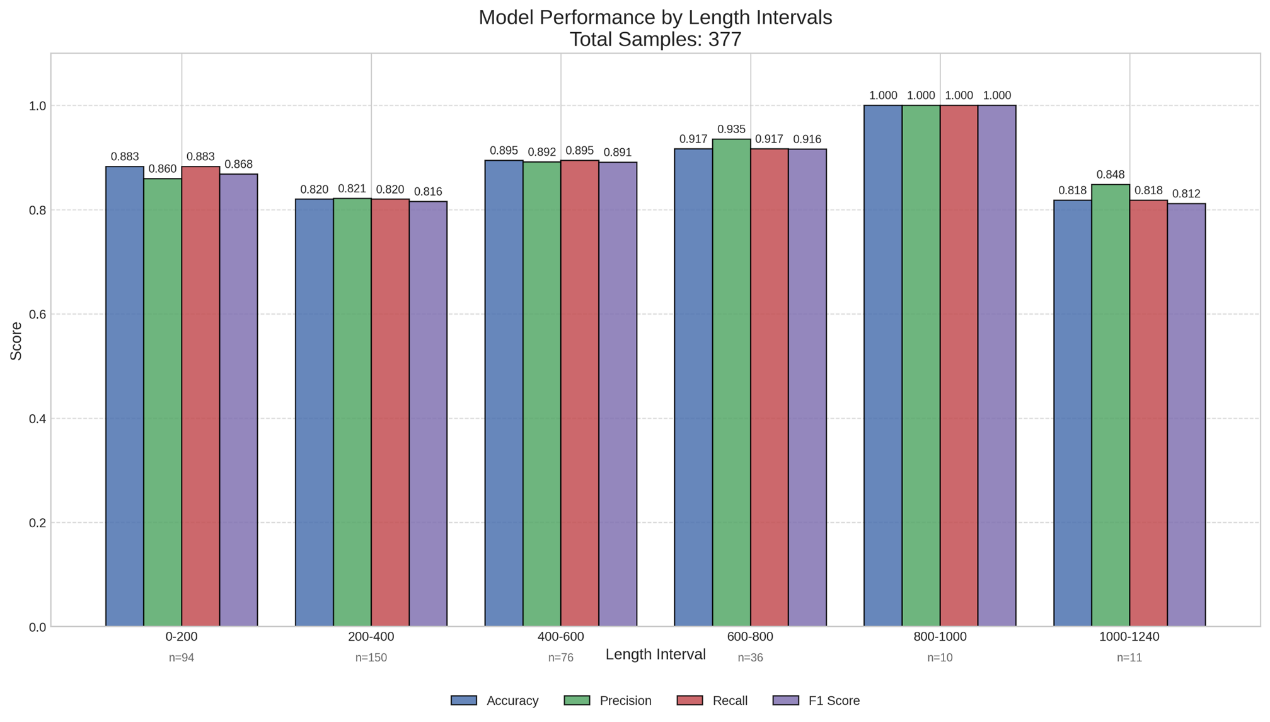


**S3 Fig. PLM-GNN’s performance across length intervals.**


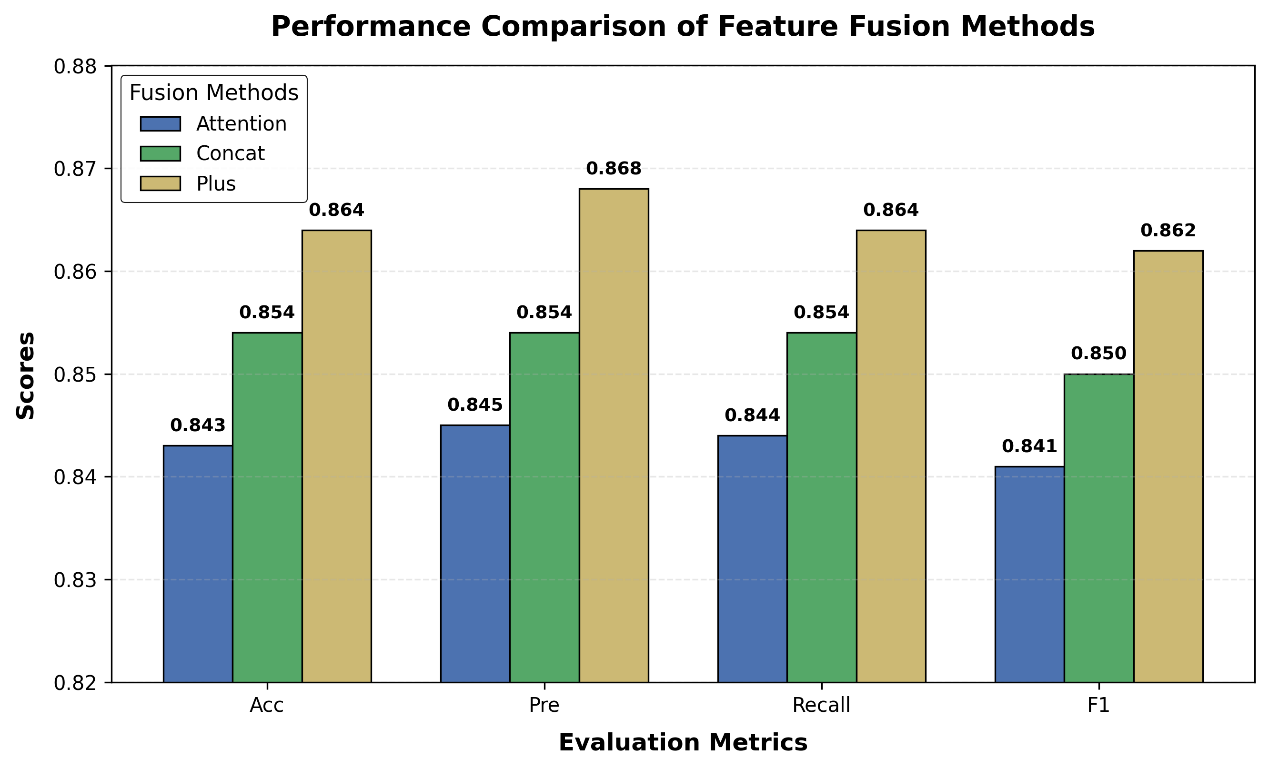


**S4 Fig. Performance of PLM-GNN under different feature fusion strategies.**


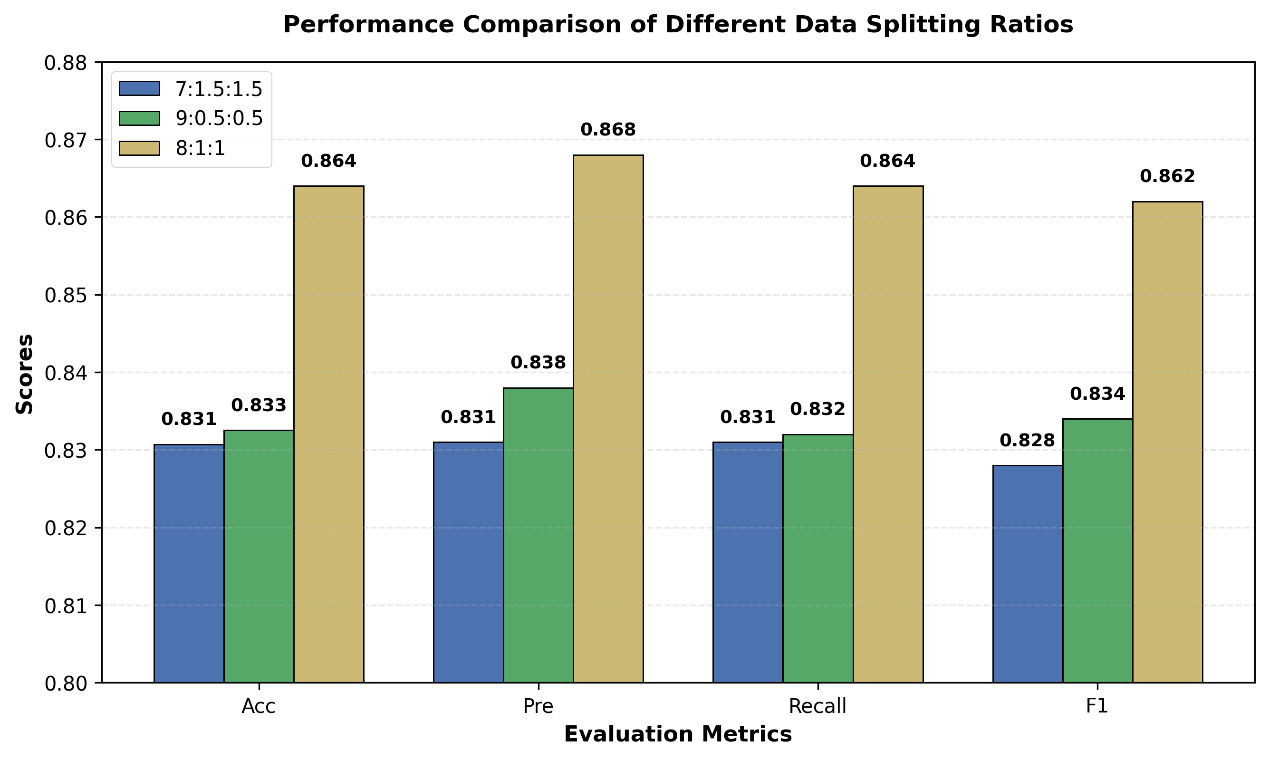


**S5 Fig. Performance of PLM-GNN under different data splits.**

**Supplemental Tables**

**S1 Table. The detailed setting information for the comparison methods. (Head denotes the number of attention heads, Hiddendim denotes the dimension of the hidden layer, Lr denotes the learning rate value, and Layer denotes the number of layers.)**

| Methods | Parameters |  |
| --- | --- | --- |
| GAT | Head: [2, 4, 6, **8**]  Hiddendim: [**128**, 256]  Lr: [1e-5, 1e-4, **1e-3**, 1e-2]  Laryer: [**1**, 2] | Loss = “WeightedCrossEntropyLoss”, Optimizer = “AdamW”,  Prediction_activation = “softmax” |
| GCN | Hiddendim: [**128**, 256]  Lr: [1e-5, **1e-4**, 1e-3, 1e-2]  Laryer: [1, **2**] |  |
| GraphTransformer | Head: [2, 4, 6, **8**]  Hiddendim: [128, **256**]  Lr: [1e-5 ,**1e-4**, 1e-3, 1e-2] |  |
| CNN | Hiddendim: [**128**, 256]  Lr: [1e-5, **1e-4**, 1e-3, 1e-2]  Laryer: [1, **2**] |  |
| BiLSTM | Hiddendim: [128, **256**]  Lr: [1e-5, 1e-4, **1e-3**, 1e-2] |  |
| GRU | Hiddendim: [128, **256**]  Lr: [1e-5, 1e-4, **1e-3**, 1e-2] |  |
| LSTM | Hiddendim: [**128**, 256]  Lr: [1e-5, 1e-4, **1e-3**, 1e-2] |  |
| Transformer | Head: [2, 4, 6, **8**]  Hiddendim: [128, **256**]  Lr: [1e-5, **1e-4**, 1e-3, 1e-2] |  |
| CNN-LSTM | Hiddendim: [128, **256**]  Lr: [1e-5, **1e-4**, 1e-3, 1e-2] |  |
| CNN-BiLSTM | Hiddendim: [128, **256**]  Lr: [1e-5, 1e-4, 1e-3, **1e-2**] |  |
| CNN-GRU | Hiddendim: [128, **256**]  Lr: [1e-5, 1e-4, **1e-3**, 1e-2] |  |
