# Supplementary material for: Classification of virulence factors based on dual-channel neural networks with pre-trained language models
Source: PLoS One. 2026 Jan 5;21(1):e0340194. doi: 10.1371/journal.pone.0340194 (PMC12768247; doi:10.1371/journal.pone.0340194)
Supplement: Attachment — Submitted filename: Response to Reviewers.docx [file pone.0340194.s005.docx]

Dear Editor,

On behalf of my co-authors, we thank you very much for giving us an opportunity to revise our manuscript. We are truly grateful to the comments and suggestions from you and the reviewers. Those comments are very helpful for revising and improving our paper. We have studied comments carefully and tried our best to revise and improve the manuscript and made great changes in the manuscript according to the reviewers’ comments. Revised portions are marked in yellow in the paper. The main corrections in the paper and the responses to the reviewer’s comments are listed as following:

**Reviewer #1**

1. **Comment:** **A critical missing element is the evaluation of the quality of the predicted 3D structures from ESMFold. The entire structure-based channel relies on these predictions. The authors should report the predicted Local Distance Difference Test (pLDDT) scores for the dataset. It is crucial to analyze if there is a correlation between low-confidence structure predictions and model misclassifications. A model trained on inaccurate or low-confidence structures may learn artifacts rather than true biological signals.**

**Response:** We thank the reviewer for raising this critical point regarding the quality of ESMFold-predicted structures and its relationship with the reliability of our structure-based predictions. We fully agree that assessing prediction confidence is essential for interpreting model outputs. In response to this comment, we have now included a comprehensive evaluation of the pLDDT scores across our dataset and explicitly examined their correlation with model performance.

We computed the mean pLDDT per protein as an overall confidence metric. As shown in S1 File’s S1 Fig, the global distribution of pLDDT values is right-skewed, with a mean of 76.46. The majority of residues fall into medium- to high-confidence intervals (70–90: 54.3%; >90: 18.9%), though a proportion of low-confidence predictions (<50) remains (10.0%). We also observed certain variations across categories; for instance, “*Biofilm*” and “*Nutritional/Metabolic factor*” categories showed high mean confidence (84.7 and 84.9, respectively), while “*Effector delivery system*” had lower confidence (mean = 70.1).

Most importantly, we directly assessed whether low-confidence predictions correlate with misclassifications by our model (PLM-GNN). We computed both Pearson and Spearman correlation coefficients between the mean pLDDT scores and model classification errors. The results show no significant correlation (Pearson p = 0.1519; Spearman p = 0.0987), indicating that our model exhibits strong robustness. We hypothesize that this may be attributed to the overall high pLDDT values in the dataset, with a small number of low-pLDDT samples having minimal impact on the overall model performance.

**S1 Fig**. **Multi-perspective statistical analysis of pLDDT scores.**

These analyses have been added to the S1 File “Distribution of pLDDT and Correlation with Model Performance”. We acknowledge that some regions exhibit low confidence and appreciate the reviewer’s emphasis on careful interpretation. Still, the absence of a significant correlation suggests that our model does not systematically rely on low-quality structural artifacts for classification.

Thank you again for this insightful suggestion, which has strengthened our evaluation.

2. **Comment: The manuscript states that VFs longer than 1240 amino acids were excluded due to GPU memory limitations. This is a significant constraint that could introduce bias. The authors should provide a more precise quantification of what percentage of the total VFs in the initial dataset this exclusion represents. Furthermore, a discussion is needed on the potential biological implications, as longer proteins may possess complex domain architectures or functions that the model is now unable to learn.**

**Response:** We thank the reviewer for this insightful comment regarding the exclusion of longer VFs and its potential implications. We agree that this filtering step may introduce bias, and we have now provided further clarification and quantification in the revised manuscript.

Specifically, the initial dataset contained 4,020 VF sequences. After removing sequences with >90% homology using CD-HIT, 3,880 sequences remained. Among these, 128 sequences (3.29% of the dataset) were longer than 1240 amino acids and were excluded due to GPU memory constraints. We have explicitly stated these proportions in the Methods section’s subsection “Dataset description” (lines 167-172) for clarity.

We fully acknowledge that the exclusion of longer proteins may limit the model's ability to learn from complex, multi-domain VFs, which are often associated with sophisticated mechanisms. To better contextualize this limitation, we included an analysis of the length distribution of VFs across categories (now in Fig 2). This analysis confirms that long sequences are rare and non-uniformly distributed among classes.

**Fig 2. The length distribution of various types of VFs.**

Nevertheless, given that such long sequences constitute a very small fraction (3.29%) of the overall dataset, we expect that their exclusion has not substantially skewed the model’s general representativeness or performance. In the Discussion and conclusion section(lines 659–663), we have further reflected on this limitation and stated plans for future work to overcome computational constraints—such as employing larger-memory GPUs or developing a segmenting strategy—to include all VFs regardless of length.

Thank you again for raising this important point, which has helped us improve the clarity and rigor of our manuscript.

3. **Comment: The model's dataset is limited to the seven most numerous VF categories, excluding seven rarer ones. While this is a practical decision for model training, it limits the claimed generalizability of the model. The authors should be more explicit about this limitation and frame their conclusions accordingly, acknowledging that the model's utility for less common VF types is currently unknown.**

**Response:** We thank the reviewer for this valuable comment. We fully agree that the restriction of our training dataset to the seven most abundant VF categories represents a significant limitation to the claimed generalizability of PLM-GNN.

In the revised manuscript, we have explicitly stated in the Discussion and conclusion section (lines 652–656) that our model was trained and evaluated only on the majority categories and that its performance on the seven excluded, rarer VF categories remains entirely unknown. This limitation unavoidably constrains the model's applicability and predictive power for under-represented VF types. We have therefore reframed our conclusions to more cautiously reflect that the current model is validated primarily for common VF categories, and any broader claims about generalizability across all VF types would be premature.

To address this in future work, we plan to actively incorporate samples from these rare categories, possibly employing few-shot learning or data augmentation techniques, to build a more comprehensive and universally applicable model. We appreciate the reviewer’s emphasis on this point, which helps provide a more accurate interpretation of our results.

4. **Comment: The method for fusing the sequence and structure channel embeddings is a simple summation. This architectural choice requires justification. The authors should explore and compare this with other fusion strategies, such as concatenation followed by a linear layer or an attention-based fusion mechanism, to demonstrate that summation is indeed an optimal or sufficient choice.**

**Response:** We thank the reviewer for raising this important question regarding our feature fusion strategy. We fully agree that the choice of fusion method is a critical architectural decision that requires thorough justification.

In response to this comment, we systematically explored and compared three distinct fusion strategies: (1) direct addition, (2) concatenation, and (3) adaptive weighted fusion based on an attention mechanism. As shown in S3 File’s S4 Fig of the Supplementary Information, our comparative analysis demonstrated that the direct addition strategy achieved the best performance on the validation set for the VF classification task. Although we initially anticipated that more complex mechanisms such as attention might outperform simpler methods, the additive fusion consistently delivered superior results in our experiments. We hypothesize that this may be because the feature embeddings from the sequence and structure channels are already well-aligned in the same latent space, allowing their direct summation to serve as an effective and efficient means of feature integration.

**S4 Fig. Performance of PLM-GNN under different feature fusion strategies.**

We have now expanded the description of the fusion strategy comparison in the Methods section’s subsection“Classifying VFs and weighted cross-entropy loss”(lines 328-339) to make the rationale for this choice more explicit. We also include a brief discussion of the experimental results—noting that while additive fusion proved optimal for our specific model and dataset, the best strategy may vary for other tasks, and the choice of fusion method should always be validated empirically.

Thank you for prompting us to clarify this essential aspect of our model architecture.

5. **Comment: The analysis of remote homology detection is compelling but appears anecdotal. To strengthen this claim, the authors should perform a systematic analysis. They could identify all pairs in the test set that qualify as remote homologs (e.g., sequence identity < 30%, TM-score > 0.5) and report the model's performance specifically on this subset, rather than presenting only two cherry-picked examples.**

**Response:** We thank the reviewer for this insightful suggestion. We agree that a more systematic evaluation is essential to robustly demonstrate the model's capability in detecting remote homology.

In response to this comment, we have now conducted a comprehensive analysis to quantitatively evaluate the model’s performance on remote homologs. We systematically identified all protein pairs between the training and test sets that meet the stringent criteria for remote homology (sequence identity < 30% and TM-score > 0.5). This process resulted in a dedicated evaluation subset comprising 93 test samples, all of which are remote homologs of sequences in the training set. PLM-GNN achieved an accuracy of 91.4% on this challenging subset (85 out of 93 samples predicted correctly). This performance significantly surpasses that of the sequence channel (79 out of 93 correct) and the structure channel (70 out of 93 correct), highlighting the critical importance of integrating both sequence and structural information for recognizing distantly related analogs. We have added this systematic analysis to the Results section’s subsection “Accurately classifying VFs with remote homology”(lines 623-635) and incorporated the quantitative results into the revised manuscript.

Thank you for this valuable suggestion, which has greatly enhanced the persuasiveness of our study.

6. **Comment: When comparing PLM-GNN with other models (Table 3), it is not specified whether the hyperparameters for these baseline models were also rigorously optimized. For a fair comparison, all competing models should be tuned to their best performance on the validation set, just as the proposed model was. The authors should clarify their procedure for this.**

**Response:** We thank the reviewer for their important comments regarding the hyperparameter optimization of the baseline models, which are essential for ensuring a fair comparison.

We have re-optimized the parameters for all baseline models and evaluated their performance on the test set using the hyperparameter configurations that achieved the best performance on the validation set. All baseline models listed in S3 File’s Table S1 of the supplementary information underwent this tuning process. For each type of baseline method, we conducted a systematic grid search within the hyperparameter ranges provided in S3 File’s Table S1, covering key architectural and training parameters such as the number of attention heads (for attention-based models), hidden layer dimensionality, learning rate, and number of layers. Each model configuration was evaluated on the same validation set used for PLM‑GNN, and the best-performing configuration was selected for the final comparison in Table 3. Additionally, Table 3 and Fig 6 in the main text have been updated accordingly.

Table S1. The detailed setting information for the comparison methods. (Head denotes the number of attention heads, Hiddendim denotes the dimension of the hidden layer, Lr denotes the learning rate value, and Layer denotes the number of layers.)

| Methods | Parameters |  |
| --- | --- | --- |
| GAT | Head: [2, 4, 6, **8**]  Hiddendim: [**128**, 256]  Lr: [1e-5, 1e-4, **1e-3**, 1e-2]  Laryer: [**1**, 2] | Loss = “WeightedCrossEntropyLoss”, Optimizer = “AdamW”,  Prediction_activation = “softmax” |
| GCN | Hiddendim: [**128**, 256]  Lr: [1e-5, **1e-4**, 1e-3, 1e-2]  Laryer: [1, **2**] |  |
| GraphTransformer | Head: [2, 4, 6, **8**]  Hiddendim: [128, **256**]  Lr: [1e-5 ,**1e-4**, 1e-3, 1e-2] |  |
| CNN | Hiddendim: [**128**, 256]  Lr: [1e-5, **1e-4**, 1e-3, 1e-2]  Laryer: [1, **2**] |  |
| BiLSTM | Hiddendim: [128, **256**]  Lr: [1e-5, 1e-4, **1e-3**, 1e-2] |  |
| GRU | Hiddendim: [128, **256**]  Lr: [1e-5, 1e-4, **1e-3**, 1e-2] |  |
| LSTM | Hiddendim: [**128**, 256]  Lr: [1e-5, 1e-4, **1e-3**, 1e-2] |  |
| Transformer | Head: [2, 4, 6, **8**]  Hiddendim: [128, **256**]  Lr: [1e-5, **1e-4**, 1e-3, 1e-2] |  |
| CNN-LSTM | Hiddendim: [128, **256**]  Lr: [1e-5, **1e-4**, 1e-3, 1e-2] |  |
| CNN-BiLSTM | Hiddendim: [128, **256**]  Lr: [1e-5, 1e-4, 1e-3, **1e-2**] |  |
| CNN-GRU | Hiddendim: [128, **256**]  Lr: [1e-5, 1e-4, **1e-3**, 1e-2] |  |

This systematic optimization process ensures that all models achieve their best possible performance on the dataset used in this study, thereby enabling a fair and meaningful comparison.

We appreciate the reviewer’s suggestion to clarify this aspect, which further enhances the credibility of our comparative analysis.

7. **Comment: While the performance improvements of PLM-GNN are shown, the authors have not reported whether these improvements are statistically significant. Statistical tests (e.g., McNemar's test or bootstrapping to generate confidence intervals for the metrics) should be performed on the performance differences between PLM-GNN and the next-best-performing models to validate the superiority of the proposed method.**

**Response:** We thank the reviewer for this insightful suggestion regarding the statistical validation of our model's performance. We agree that demonstrating the statistical significance of the improvements is essential.

In response to this comment, we have now performed a comprehensive statistical significance analysis to compare PLM-GNN against the top-performing baseline models. The test set was divided into five equally sized subsets. In each iteration, four subsets were used for evaluation, resulting in five independent performance measurements for each model. We then conducted independent samples t-tests between PLM-GNN and each of the best-performing baselines (GCN and GAT) across multiple evaluation metrics.

As shown in Fig 5, PLM-GNN demonstrated consistent and statistically significant superiority over both comparison models, with all p-values being less than 0.05. This analysis confirms that the performance improvements achieved by our model are not only consistent but also statistically significant. We have added these results to the manuscript in the Methods section’s subsection " Comparison with other methods in terms of VF classification"(page 23) to provide rigorous statistical validation of our conclusions.

**Fig 5.** **Statistical significance comparison of PLM-GNN with GCN and GAT in VF classification**

8. **Comment: The paper would benefit from a more in-depth error analysis beyond the confusion matrix. The authors should investigate the characteristics of the misclassified VFs. For example, are they more likely to have low pLDDT scores, belong to specific organisms, have lengths close to the 1240 residue cutoff, or share features with another class that explains the confusion?**

**Response:** We thank the reviewer for this insightful suggestion regarding a more comprehensive error analysis. We have conducted an in-depth investigation into the characteristics of misclassified VFs, focusing specifically on the potential influencing factors mentioned in the comment.

First, in the S1 File“Distribution of pLDDT and Correlation with Model Performance,”we computed both Pearson and Spearman correlation coefficients between the misprediction label vector of the PLM-GNN and the pLDDT score vector. The results indicated no significant correlation, suggesting that model errors are not attributable to low-confidence structure predictions. Next, we examined whether misclassifications were concentrated in specific organisms. As shown in S3 File’s S2 Fig (Error rate heatmap of bacterial genera by VF category), no particular genus exhibited significantly elevated error rates, indicating that taxonomic origin is not systematically associated with misclassification. We then analyzed whether sequence length near the 1,240-residue cutoff influenced errors. Sequences were grouped into intervals (each spanning 200 residues), and performance metrics were calculated for each group. S3 File’s S3 Fig demonstrates that sequences in longer intervals (1000-1,240 residues) performed comparably to those in shorter intervals (200-400 residues), suggesting no clear relationship between sequence length and misclassification. Finally, t-SNE visualization of the fused features (Fig 7D) revealed that samples from different categories (e.g., *Immune modulation* and *Nutritional/Metabolic factor*) clustered closely in the embedding space, indicating high feature similarity. This implies that most errors occur between functionally or structurally similar categories where the model struggles to distinguish closely related features.

**S2 Fig. Error rate heatmap of bacterial genera by VF category.**

**S3 Fig. PLM-GNN’s performance across length intervals.**

These analyses have been added to the Results section’s subsection “Analysis of Potential Causes for Model Prediction Errors” (lines 544-564). Thank you for this valuable suggestion, which has significantly strengthened our error analysis section.

9. **Comment: The description of the geometric features for the graph representation (Table 2) is very dense. While comprehensive, its complexity could hinder reproducibility. A more detailed, step-by-step explanation in the supplementary materials, perhaps with a visual diagram illustrating how these features are derived from a protein structure, would greatly improve clarity.**

**Response:** We thank the reviewer for this valuable feedback regarding the complexity of our geometric feature description. We agree that a more detailed, step-by-step explanation would significantly enhance clarity and reproducibility.

In the S2 File "Detailed Derivation of Geometric Graph Features" of the Supplementary Information, we provide a comprehensive description of the feature extraction method for the geometric graph neural network (GNN). The core concept involves constructing a radius graph with *Cα* atoms of amino acid residues as nodes, connected within a 15Å cutoff, and establishing a local right-handed coordinate system for each residue defined by its backbone atoms (*N, Cα, C*). This enables the construction of a set of mathematically rich, rotation- and translation-invariant representations for both nodes and edges. The resulting geometrically informed node features incorporate four types of information: sine and cosine values of backbone dihedral and bond angles, radial basis function (RBF) encodings of distances between 10 key atom pairs, direction vectors of four key atoms in the local coordinate system, and sequence features extracted by ProtT5. The edge features integrate RBF encodings of 25 inter-residue atom pair distances, direction vectors between atoms computed from both perspectives, quaternions representing relative rotations, and positional encodings based on *Cα-Cα* vectors, collectively describing the geometric relationships between residues.

These additions provide a complete guide to reproducing our geometric feature extraction process. We believe these enhancements substantially improve the clarity and reproducibility of our method while maintaining the comprehensive nature of our feature engineering approach.

Thank you for this excellent suggestion, which has significantly improved the accessibility of our technical approach.

10. **Comment: The conclusion that the model "can provide valuable theoretical support for the development of antiviral strategies" is an overstatement. The model is a classification tool, which is an important step, but it does not directly provide mechanistic insights or drug targets. A more measured and precise conclusion regarding the model's immediate application—improving the functional annotation of proteins—would be more appropriate.**

**Response:** We sincerely thank the reviewer for this insightful comment. We agree that our original conclusion may have overstated the implications of our work. As pointed out, PLM-GNN is primarily a classification tool that improves the functional annotation of VFs, and while it may facilitate downstream studies on virulence mechanisms and therapeutic target discovery, it does not directly provide mechanistic insights or drug targets. We have carefully revised the Discussion and conclusion section (lines 646-651) to make it more precise and measured. Specifically, we now emphasize that the immediate contribution of our model lies in enhancing the accuracy of VF classification and functional annotation. The potential utility in supporting the development of anti-virulence strategies is mentioned only as a long-term perspective rather than a direct outcome of this work.

11. **Comment: In the "Applied to effector delivery system classification" section, the model's performance on the T7SS category is noted to be weaker due to the small sample size. This highlights a potential weakness in handling highly imbalanced data. The authors could discuss or experiment with advanced data augmentation techniques or few-shot learning approaches as potential future work to address this.**

**Response:** We sincerely thank the reviewer for their insightful observations regarding the model’s performance on the T7SS category and fully agree that data imbalance poses a significant challenge to minority class prediction. We greatly appreciate the suggestion to explore advanced techniques such as data augmentation and few-shot learning.

In the Discussion and conclusion section (lines 656-660), we have explicitly addressed this issue and outlined plans for improvement. Specifically, we noted that “the model underperformed in predicting minority classes such as effector delivery systems (e.g., T7SS), highlighting the challenges posed by highly imbalanced data,”and emphasized that “future work will explore solutions such as few-shot learning or employing generative adversarial networks (GANs) to generate samples for rare categories.”

We agree that these advanced approaches hold great promise for more effectively addressing class imbalance in biological data. In subsequent studies, we plan to incorporate methods such as these into our future work to enhance the model’s ability to recognize underrepresented VF categories like T7SS.

Thank you for this valuable suggestion, which will strongly facilitate our further exploration in this research direction.

12. **Comment: The interpretability of the model is primarily explored through t-SNE visualizations. To further this, the authors could consider using model interpretability techniques like integrated gradients or attention map analysis, especially on the Transformer components, to identify which specific amino acid residues or structural motifs are most influential for classifying a given VF type. This would provide more direct biological insights.**

**Response:** We thank the reviewer for this insightful suggestion regarding the use of advanced interpretability methods. We agree that techniques such as attention map analysis can provide more direct biological insights into the model's decision-making process.

In response to this comment, we have now conducted an additional interpretability analysis using attention mechanisms from the Transformer component of our sequence channel. Specifically, we extracted attention scores for the T4SS category and identified the top 5 amino acids with the highest attention weights across sequences: phenylalanine (F), threonine (T), proline (P), serine (S), and glycine (G). These residues are biologically significant for T4SS function—F is critical in hydrophobic motifs for effector binding and transport, T and S serve as phosphorylation sites regulating assembly, while P and G contribute to structural stability of the transmembrane channel.

This analysis demonstrates that the model's attention mechanism effectively captures key functional residues involved in transport, regulation, and structural integrity. To further enhance interpretability, we will extend this approach to other VF categories and incorporate additional methods such as integrated gradients in future work, as suggested, to identify influential structural motifs and provide more comprehensive biological insights.

We have added these findings to the Results section’s subsection "Model Interpretability Analysis"(lines 528-543) in the revised manuscript. Thank you for this valuable suggestion.

13. **Comment: For a more comprehensive comparison, further studies involving standalone Convolutional Neural Network (CNN) and Vision Transformer (ViT) models could be included. Comparing the proposed method against other sophisticated, single-modality architectures would better highlight the specific advantages of the dual-channel approach. "Hybrid deep learning model for automated colorectal cancer detection using local and global feature extraction","InceptionNeXt-Transformer: A novel multi-scale deep feature learning architecture for multimodal breast cancer diagnosis"**

**Response:** We thank the reviewer for the insightful suggestion regarding comparisons with additional single-modality architectures. We would like to highlight that our study already includes comprehensive comparisons with standalone CNN, Transformer, and Graph Transformer models (as shown in Table 3), demonstrating the clear advantage of our dual-channel approach over these single-modality alternatives.

Specifically, our experiments show that neither pure CNN-based nor Transformer-based models can match the performance of PLM-GNN, particularly in capturing both sequence patterns and structural features essential for accurate VF classification. These results align with findings from previous studies such as the "Hybrid deep learning model for automated colorectal cancer detection using local and global feature extraction" and "InceptionNeXt-Transformer: A novel multi-scale deep feature learning architecture for multimodal breast cancer diagnosis", both of which we have cited in our manuscript [52,53] to further support the importance of multi-modal integration.

We fully agree with the reviewer that extended comparisons could provide additional insights. Thank you again for this valuable suggestion, which helps strengthen the overall validation of our approach.

**Reviewer #2**

**Major Revisions:**

1. **Comment: The author should add the justification for the 8:1:1 stratified split. The paper states:“We then randomly divided the data into three subsets using an 8:1:1 ratio (with stratified sampling).”**

**Response:** We thank the reviewer for this insightful comment regarding the justification of our data splitting strategy. We agree that providing a clear rationale for the chosen 8:1:1 ratio with stratified sampling is essential.

In our study, we compared three different splitting ratios—7.5:1.5:1.5, 8:1:1, and 9:0.5:0.5—to determine the optimal partitioning strategy for our dataset. The performance of the model under each ratio was rigorously evaluated, and the 8:1:1 ratio consistently yielded the best results in terms of classification accuracy and stability in multi-class task, as illustrated in S3 File’s S5 Fig. This ratio provided a balanced compromise between sufficient training data and adequate validation/testing samples, thereby minimizing overfitting while ensuring robust evaluation.

**S5 Fig. Performance of PLM-GNN under different data splits.**

To maintain the representativeness of the original dataset—especially for minority classes—we applied stratified sampling during the splitting process, as detailed in the Methods section’s "Dataset description" subsection (lines 173-186). This ensured that the distribution of VF categories in the training, validation, and test sets remained consistent with the original dataset, preventing underrepresentation or exclusion of rare classes in any subset.

Thank you for highlighting the need for this clarification, which has improved the transparency of our methodology.

2. **The manuscript relies heavily on undefined acronyms (e.g., “CNN”, “SVM”, “Cα”). Please define each acronym at its first occurrence.**

**Response:** We thank the reviewer for pointing out the need for clearer definitions of acronyms throughout the manuscript. We sincerely apologize for this oversight.

In the revised version, we have carefully reviewed the entire text and ensured that every acronym is explicitly defined at its first occurrence.

Thank you for this helpful suggestion, which has significantly improved the clarity and accessibility of our manuscript.

3. **Several symbols appear without explanation, like (X(0)∈RL×1280X^{(0)} ).For each symbol, please include a brief textual definition at the point of first use.**

**Response:** We thank the reviewer for pointing out the need for clarification regarding mathematical symbols in the manuscript. We sincerely apologize for this oversight.

In the revised version, we have carefully added explicit textual explanations for all mathematical symbols at their first occurrence. Similar definitions have been provided for all other mathematical symbols throughout the manuscript.

Thank you for this valuable suggestion, which has significantly improved the clarity and readability of our mathematical descriptions.

We would like to thank you for allowing us to resubmit a revised version of the manuscript, and hope that the corrections will meet with approval.

We look forward to your positive response.

Yours sincerely,

Guanghui Li, PhD

Associate Professor, East China Jiaotong University

Cheng Liang, PhD

Associate Professor, Shandong Normal University
